# Gender blind? An analysis of global public-private partnerships for health

**DOI:** 10.1186/s12992-017-0249-1

**Published:** 2017-05-12

**Authors:** Sarah Hawkes, Kent Buse, Anuj Kapilashrami

**Affiliations:** 1 Institute for Global Health, University College London, 30, Guilford Street, London, WC1N 1EH UK; 2 UNAIDS, Geneva, Switzerland; 3 Global Public Health Unit, University of Edinburgh, Edinburgh, UK

**Keywords:** Global public private partnerships for health, Gender, Mainstreaming, Non-communicable diseases

## Abstract

**Background:**

The Global Public Private Partnerships for Health (GPPPH) constitute an increasingly central part of the global health architecture and carry both financial and normative power. Gender is an important determinant of health status, influencing differences in exposure to health determinants, health behaviours, and the response of the health system.

We identified 18 GPPPH - defined as global institutions with a formal governance mechanism which includes both public and private for-profit sector actors – and conducted a gender analysis of each.

**Results:**

Gender was poorly mainstreamed through the institutional functioning of the partnerships. Half of these partnerships had no mention of gender in their overall institutional strategy and only three partnerships had a specific gender strategy. Fifteen governing bodies had more men than women – up to a ratio of 5:1. Very few partnerships reported sex-disaggregated data in their annual reports or coverage/impact results. The majority of partnerships focused their work on maternal and child health and infectious and communicable diseases – none addressed non-communicable diseases (NCDs) directly, despite the strong role that gender plays in determining risk for the major NCD burdens.

**Conclusions:**

We propose two areas of action in response to these findings. First, GPPPH need to become serious in how they “do” gender; it needs to be mainstreamed through the regular activities, deliverables and systems of accountability. Second, the entire global health community needs to pay greater attention to tackling the major burden of NCDs, including addressing the gendered nature of risk. Given the inherent conflicts of interest in tackling the determinants of many NCDs, it is debatable whether the emergent GPPPH model will be an appropriate one for addressing NCDs.

## Background

Gender is an important determinant of health status – influencing, at a minimum, rates of risk-exposure to common drivers of ill-health, health-care seeking patterns, and the nature of the health system’s response to illness. It is with the latter that we are concerned in this paper. We focus on global public-private partnerships for health (GPPPH) since they are an important component of the global health architecture, are seen as having significantly increased the resources available for global health [1–4], and are promoted by some as offering critical opportunities to improve “efficiency, equity, value for money, and outcomes in global public health” [5]. Moreover, the public-private partnership approach has been encouraged as a mechanism for overcoming failures of both market and state [6] as well promoting efficiency in service delivery [6] and innovation in and access to technological resources [7]. Given the importance of the GPPPH model within global health activities, and the central role that gender plays as a determinant of health, the question we address in our paper is how and to what extent the GPPPH focus on issues of gender within their priorities, policies and programmes.

The GPPPH have become increasingly powerful actors within global health: funds allocated to the two largest GPPPH (Global Fund to Fight AIDS, TB and Malaria –GFATM- and GAVI) have increased from US$1.67 billion to US$4.9 billion over the 10-year period 2005 and 2015 – an almost three-fold rise. In total, these two partnerships currently receive 14% of external global health financing [8]. Nonetheless, the partnership model has been criticised as implying privatisation by stealth [9], of disrupting the focus and governance of country level health systems [10–12], of unduly influencing international norm-setting [13, 14] and of promoting an overly narrow technical focus on solutions for health problems [15, 16] . Notwithstanding such criticism, public-private-interaction is likely to grow across all sectors, including health, as countries and international organisations mobilise to achieve the sustainable development goals (SDG) by 2030. The United Nations Agenda 2030 for Sustainable Development actively advocates for countries to “Encourage and promote effective public, public-private and civil society partnerships, building on the experience and resourcing strategies of partnerships.” [17] . Moreover, the Addis Ababa Agenda on Financing For Development identifies a critical role of private finance for development, including through the mechanism of public-private partnerships [18].

Although there are no agreed upon definitions of GPPPH [19] a commonly used definition was proposed by Buse and Harmer as “relatively institutionalised initiatives, established to address global health problems, in which public and for-profit private sector organisations have a voice in collective decision-making.” [20] We use this definition for identifying GPPPH for inclusion in our analysis.

Gender is recognised as a significant driver of health outcomes – both as an influence in its own right, and through its interaction with other determinants of inequity and vulnerability. Payne [21] has characterized gender influences on health in three domains: differences in exposure to social determinants of health (e.g., poverty or the health risks of employment); health behaviours (e.g., diet, tobacco or alcohol use, patterns of care seeking); and the response of the health system to the different needs of men and women.

Despite the profound influence that gender exerts on health, the ability of global health institutions to recognise, understand and address the influence of gender on health outcomes has been characterised as “missing, misunderstood and sometimes mainstreamed” [22]. Gender mainstreaming - a strategy for promoting gender equity through “research, legislation, policy development and in activities on the ground” [23] - has been part of global policy discourse for more than 35 years [24] and the United Nations (UN) Economic and Social Council agreed to mainstream a gender perspective in all UN policies and programmes with the ultimate goal of gender equality [25] . The concept and practice of gender mainstreaming is contested. Critics in particular emphasise how an ‘integrationist’ and ‘technocratic’ approach adopted by state and international bureaucracies, waters down its transformative intent [26]. Yet, the contributions of GM in placing gender as “a critical axis of power” (ibid) and improving effectiveness of policies “by making visible the gendered nature of assumptions, processes and outcomes” [27] are widely acknowledged.

Reviews of how international health organisations “do gender” have predominantly focused on major intergovernmental agencies [21] and to date there have been few evaluations of how the GPPPH take gender equity into account when prioritising the focus of their work, their governance structures, or their ways of implementing activities. Hanefeld et al. reviewed the impact of three global health initiatives on health equity, with a specific focus on HIV and women, but concluded that there was little evidence of long-term impact on equity [28]. More recently, Gideon and Porter reviewed public-private partnerships with an emphasis on women’s health, and noted the lack of evaluation of partnerships with respect to their impact on various aspects of equity, including gender [29].

Within this paper we take a holistic approach to gender equity in health and analyse how the GPPPH address gender– both as a determinant of health, and as an influence on health system priorities. We use the methodology of gender analysis to systematically identify and critically appraise gender policies and commitments of GHPPP to assess key gaps and address gender related health inequities [30].

## Methods

We included 18 GPPPH that fit the definition of Buse and Harmer [20]. The partnerships were identified from previous publications, WHO documents on partnerships and collaboration [31], and from email communications with experts in public private partnerships, global health and global health governance. We also posted a question on Twitter to try and identify partnerships fitting the Buse/Harmer definition (and identified one GPPPH this way). We did not include public-private partnerships that operate only at national (or sub-national) levels, or those partnerships that do not have a formal governance mechanism that includes both the public sector and the for-profit private sector.

We conducted a gender analysis of the priorities, focus and institutional mechanisms of each of the 18 GPPPH. The analysis relied upon reviewing the publicly-available documents from each partnership found on their websites. We analysed the following: references to or working definition of gender used by the GPPPH; presence of a specific gender strategy; gender breakdown of the governing board; presentation of gender-disaggregated service delivery and/or health outcome and impact data. We reviewed the make-up of the governing boards, and categorised board members as belonging to: the public sector, i.e., national government or multilateral system; the private for-profit sector; or “other” – i.e., academics, representatives of Foundations, non-governmental or civil society organisations, consultants (including now retired staff from other sectors), and people whose affiliations were not fully clear.^1^ In some cases, people shifted their position from representing public to private sectors (occasionally, vice-versa) as they changed jobs; we categorized them according to their current official position.

**Fig. 1:**
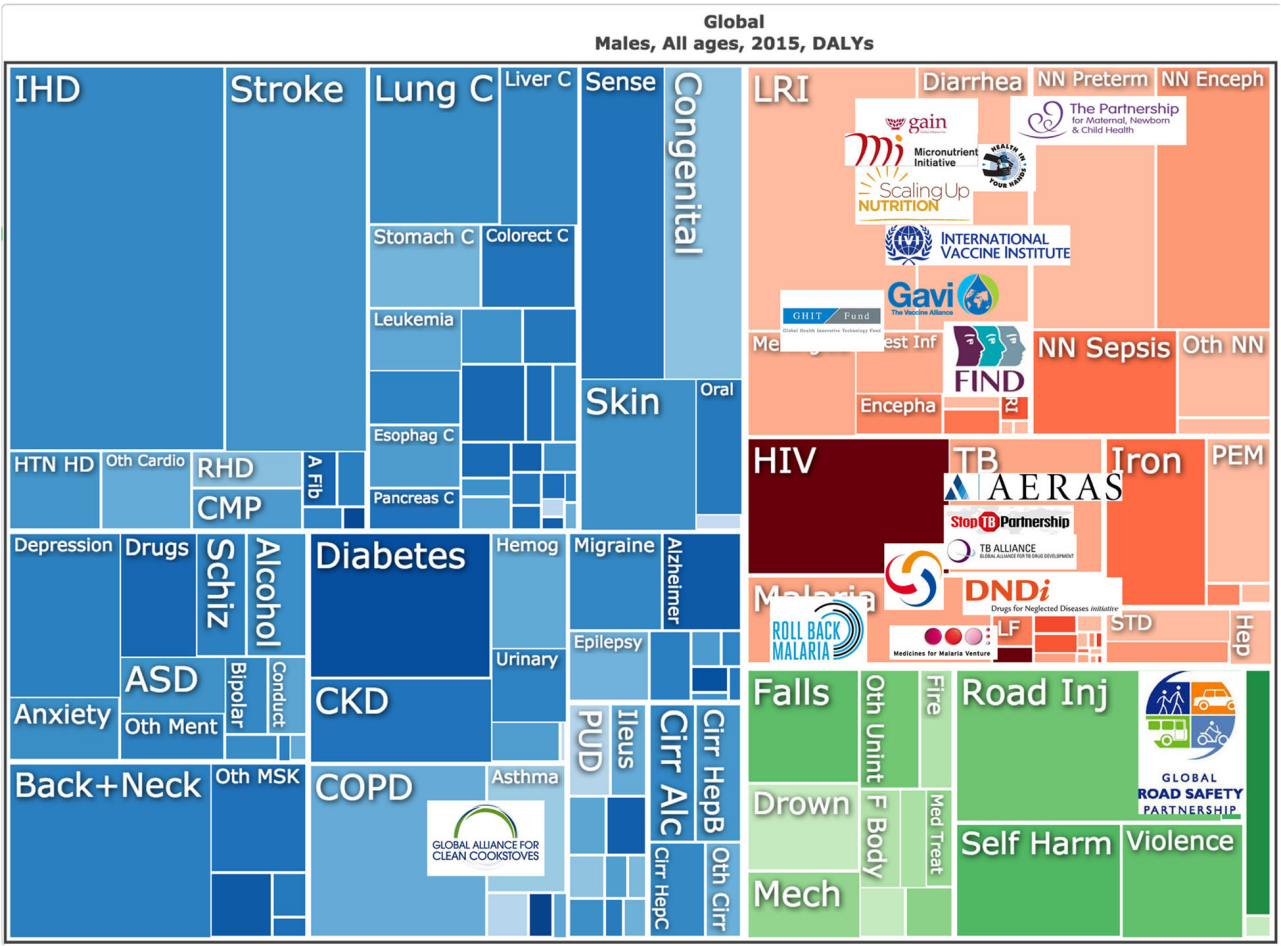
DALY distribution and GPPPH area of work; men. Data and chart available at: https://vizhub.healthdata.org/gbd-compare/

Our methodological approach to analyzing the extent to which gender is mainstreamed within health institutions predominantly focused on the extent to which a gender perspective is incorporated into organizational governance, structure and policies [32, 33]. Moving beyond this established approach, we were additionally concerned with the extent to which gender played a part in institutional priorities – were the GPPPH addressing the impact of gender on the overall illness burden? As a final methodological step, we compared the focus of the 18 GPPPH with the current evidence of global burden of disease, disaggregated by sex, and reviewed whether the GPPPH address the main determinants of morbidity, mortality and well-being. In Figs. 1 (men) and 2 (women) we mapped GPPPH focus on to the global burden of disease charts produced by the Institute for Health Metrics and Evaluation [34]. These charts show the proportional distribution of burden of disease, as measured by disability adjusted life years (DALYs), and are divided (by IHME) into three categories: non-communicable diseases including mental health (left hand side of Figure); maternal and communicable diseases (top right); and violence and injury (bottom right). The logos of each GPPPH are mapped onto the burden of disease charts. Exact mapping was not possible since many of the partnerships cover several health problems (e.g., developing new diagnostics for infectious diseases, delivering vaccines against infection, or improving maternal health), but the placement of partnership logos gives a general idea of the main areas of work.

**Fig. 2:**
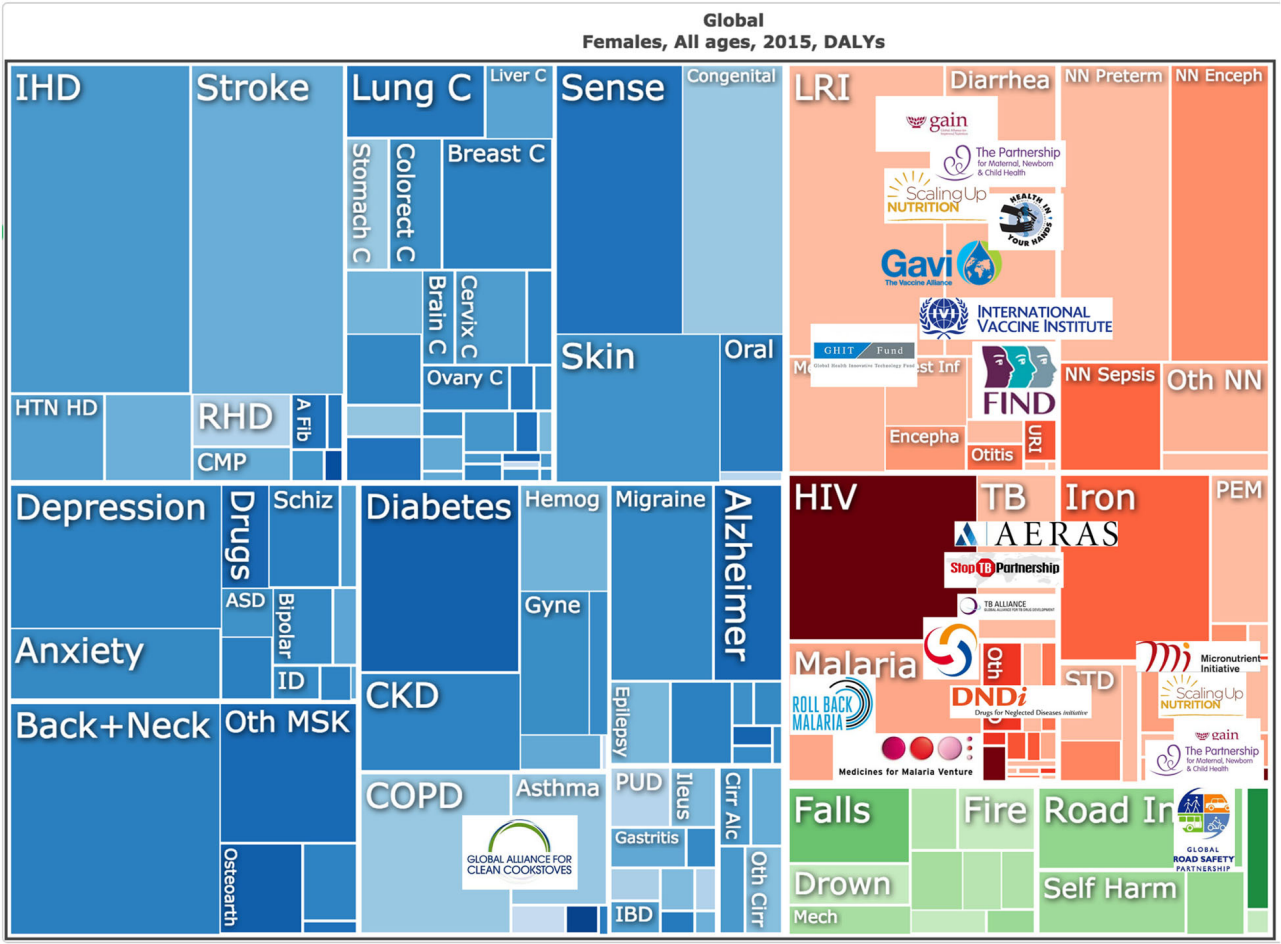
DALY distribution and GPPPH area of work; women. Data and chart available at: https://vizhub.healthdata.org/gbd-compare/

## Results

A full list of these GPPPH is given in Table 1 along with their main focus area of work – mainly covering product development and access; strengthening systems including health systems; or addressing the underlying determinants of ill-health.

**Table 1:** general focus of each partnership included in the analysis

Global Health Public-Private Partnership	Website address	Mission
AERAS	http://www.aeras.org/	To develop new, effective TB vaccines that are affordable and accessible to all who need them.
DNDi	http://www.dndi.org/	To develop new drugs or new formulations of existing drugs for people living with neglected diseases.
FIND diagnostics	http://www.finddx.org/	To turn complex diagnostic challenges into simple solutions to overcome diseases of poverty and transform lives.
gain	http://www.gainhealth.org/	To reduce malnutrition through sustainable market-based strategies aimed at improving the health and nutrition of populations at risk.
GAVI	http://www.gavi.org/	To save children’s lives and to protect people’s health by increasing equitable use of vaccines in lower-income countries.
Global Health Innovative Technology Fund	https://www.ghitfund.org/	To facilitate international partnerships that bring Japanese innovation, investment, and leadership to the global fight against infectious diseases and poverty in the developing world.
Global Road Safety Partnership	http://www.grsproadsafety.org/	To dedicate its partnership to the sustainable reduction of road-crash death and injury in low- and middle-income countries, which suffer 90 per cent of the 1.3 million annual deaths and 50 million serious injuries that arise from road crashes.
International Vaccine Institute	http://www.ivi.int/	To discover, develop, and deliver safe, effective and affordable vaccines for global public health.
Medicines for Malaria Venture	http://www.mmv.org/	To reduce the burden of malaria in disease-endemic countries by discovering, developing and facilitating delivery of new, effective and affordable antimalarial drugs.
Roll Back Malaria	http://www.rollbackmalaria.org/	To reduce malaria morbidity and mortality by reaching universal coverage and strengthening health systems.
Scaling Up Nutrition	http://scalingupnutrition.org/	To ensure high quality and tailored support for efforts to scale up nutrition in line with both national and global targets (e.g., the 6 targets set at the 2012 World Health Assembly).
Stop TB Partnership	http://www.stoptb.org/	To serve every person who is vulnerable to TB and ensure that high-quality diagnosis, treatment and care is available to all who need it.
TB Alliance	http://www.tballiance.org/	To dedicate its organisation to the discovery and development of better, faster-acting, and affordable TB drugs that are available to those who need them.
The Global Alliance for Clean Cookstoves	http://cleancookstoves.org/	To save lives, improve livelihoods, empower women, and protect the environment by creating a thriving global market for clean and efficient household cooking solutions.
The Global Fund	http://www.theglobalfund.org/en/	To attract, manage and disburse additional resources through a new PPP that will make a sustainable and significant contribution to the reduction of infections, illness and death, thereby mitigating the impact caused by HIV/AIDS, TB and malaria in countries in need, and contributing to poverty reduction as part of the MDGs.
The Global Public-Private Partnership for Handwashing	http://globalhandwashing.org/	To drive forward, develop, and share knowledge to strengthen handwashing implementation, build political commitment, and trigger action to promote handwashing at local, national, and international levels, including through advoacy initiatives, such as Global Handwashing Day.
The Micronutrient Initiative (Nutrition International)	http://www.nutritionintl.org/	To be a global leader in advancing integrated, innovative and sustainable solutions to reduce vitamin and mineral deficiencies through advocacy, technical and programmatic support, in collabortion with others.
The Partnership for Maternal, Newborn and Child Health	http://www.who.int/pmnch/en/	To increase the engagement, alignment and accountability of partners, by creating a multi-stakeholder platform that will support the successful implementation of the Global Strategy for Women’s, Children’s and Adolescent’s Health, enabling partners to achieve more together than any individual Partner could do alone.

Table 2 presents the results of the gender analysis of the 18 GPPPH. Three partnerships (GAVI, Global Fund for AIDS, TB and Malaria – The Global Fund - and Global Alliance for Clean Cookstoves) had a specific gender strategy, although the understanding of gender varied significantly across the three. For GAVI, gender was defined as “the characteristics of women and men which are socially constructed” while the Global Alliance for Clean Cookstoves emphasised the need to focus their work on girls and women. The Global Fund in its Gender Equality Strategy [35] commits to fund proposals “that scale up services and interventions that reduce gender-related risks and vulnerabilities to infection … and address structural inequalities and discrimination”. The Global Fund was the only partnership to highlight the specific needs of transgender populations (see Table 2) in a policy document, although the Stop TB/UNAIDS Gender Assessment Tool includes transgender health issues as a focus area.

**Table 2:** Institutional approach to gender within each GPPPH (Includes: definition of gender, presence of gender strategy, gender breakdown on governing board, gender disaggregated reporting)

Global Health Public Private Partnership	Gender strategy present?	How is gender defined or addressed?	How is gender disaggregated in monitoring and evaluation?	Male: Female representation on governing board
Product Development and Access				
AERAS	✕	✕	✕	6:4
DNDi	✕	✕	✕	8:4
FIND diagnostics	✕	✕	✕	9:2
International Vaccine Institute	✕	✕	✕	8:3
Medicines for Malaria Venture	✕	✕	✕	10:3
TB Alliance	✕	✕	✕	7:2
Addressing drivers of ill-health and injury				
GAIN	✕	Gender policy recognising impact of gender inequality on nutrition and importance of women’s empowerment to improve nutrition	✕	5:4
Global Health Innovative Technology Fund	✕	✕	✕	6:2
Global Road Safety Partnership	✕	✕	✕	10:3
Scaling Up Nutrition	Revise national plans to include issues of women empowerment and nutrition; implement existing principles at sectoral level and track equity and gender sensitive indicators; M&E plans and programs inclusive of women and marginalised groups regularly; build capacity for all participating actors to be more gender-sensitive	Women and girls = deprived groups with financial, social, structure and cultural barriers to services, without right to attain their full potential	Country level report gender balance in management boards; constitutional provisions that specifically promote women’s participation in decision making; gender-responsive policy frameworks (including legislation, policies and budgets) in planning documents; Parliaments that introduced legislative measures to promote gender equality and women’s empowerment	9:6
The Global Alliance for Clean Cookstoves	To increase role of women and address gender issues to scale adoption by (1) building evidence and sharing data; (2) building capacity of enterprises; (3) increasing access to finance; (4) raising awareness; (5) setting and influencing policies	Women and girls who breathe in harmful smoke while cooking and spend hours walking far distances to secure cooking fuel	✕	7:4 (leadership council) 4:2 (advisory council)
The Global Public-Private Partnership for Handwashing	✕	✕	✕	?
The Micronutrient Initiative (now called Nutrition International)	✕	Women’s and newborn survival and health	Additional pregnant women reached with iron and folic acid; additional people vitamin A and fortified salt	8:4
Strengthening systems				
Roll Back Malaria	✕	Multisectoral Action Framework mentions gender as a determinant that impacts malaria	✕	10:2
The Global Fund	Action Plan 2014–2016: Gender responsive programming to encourage positive bias in funding programs and activities that address gender inequalities and strengthen response for women and girls; *separate strategy in relation to sexual orientation and gender identities	Women and men, and particularly marginalised and vulnerable “key populations” such as people who use drugs, transgender people, bisexual and lesbian populations, adolescent girls, prisoners, migrants, men who have sex with men, sex workers	Will require countries to collect sex-disaggregated data and gender-sensitive information about population and ensure data available for regular analysis of gender equality approaches in health care services	15:7
The Partnership for Maternal, Newborn and Child Health	Gender transformative programming; engaging men as partners in reproductive health; men as agents of change	MDG 3. Promote gender equality and empower women	✕	12:12
Strengthening systems				
GAVI	Gender policy to scale up gender mainstreaming and promote gender equality by (1) applying gender perspective to all its work; (2) complementing partners’ efforts to promote gender equality in health; (3) promoting country ownership and alignment with regard to gender issues; (4) exercising strong leadership and demonstrating political will; Board Gender Balance (including Members and Alternate) = not more than 60% of either gender represented	Gender used to describe those characteristics of women and men which are socially constructed	Review existing sex-disaggregated data and deciding how to access and analysis, how and if can use to inform decision-making, and what additional data needed to inform appropriate vaccination strategies	15:12
Stop TB Partnership	In collaboration with UNAIDS and partners; tool to assist countries with assessing their HIV and TB epidemics and responses from a gender perspective, to ensure responses are gender-senstive with gender transformative priorities and actions	Women and girls, men and boys, and more specifically, key vulnerable populations such as sex workers, transgender people, and women and young women who use drugs	Using analysis matrix of: (1) epidemiological data; (2) social-cultural, economic and political context; (3) current HIV/TB policy response; (4) current HIV/TB programming response; idenitfy potential mismatches, gaps and opportunities and indicate how to build on existing interventions in country	13:12

X = not present

The Stop TB Partnership did not have a published gender strategy, but its overall strategy document defined gender-sensitive policies and gender-specific approaches and stressed that these should “strengthen the response to fulfil the right to health of women and girls, men and boys in all their diversity” [36] . Stop TB, jointly with UNAIDS, has recently released a gender assessment tool to assess country-level HIV and TB epidemics and guide development of gender sensitive and transformative responses [37].

A further five GPPPH (GAIN, RBM, SUN, Micronutrient Initiative and PMNCH) specifically mentioned gender and the health of women and girls in their overall strategy document. None of these partnerships included any mention of a specific focus on the health of men and boys.

Half the GPPPH (AERAS, DNDi, FIND, Global Health Innovative Technology Fund, The Global Road Safety Partnership, International Vaccine Institute, Medicines for Malaria Venture, TB Alliance and the Global PPP for Handwashing) made no mention of gender in their overall strategy, had no gender strategy, and did not allude to the specific health needs of women and girls or men and boys.

The governing bodies of identified GPPPH contained between 8 and 27 members; only one partnership (Partnership for Maternal, Newborn and Child Health) had equal numbers of women (n = 12) and men (n = 12) on its board, while Stop TB was close to parity with 12 women and 13 men. For one partnership (Handwashing) it was not possible to identify the gender breakdown of the board since the website listed organisations rather than individuals, and its secretariat did not respond to our request for information. The other 15 partnerships had governing bodies with more men than women, with a gender ratio of up to five times more men than women (Roll Back Malaria). GAVI was the only partnership with a stated policy of gender equality on its governing board, and they have 15 men and 12 women. Of note, women from the private sector were proportionally more under-represented than women in public/other sectors and ten of the 18 boards had no female private sector representation.

Very few of the partnerships reported sex- or gender-disaggregated data in their annual reports or available coverage/impact results. Since 2014, GAVI requires countries to specify when their proposals target a gender-related barrier to immunisation (specified by 39% of proposals to date) and the Scaling Up Nutrition partnership 2015 report mentions that 22 countries have gender responsive allocations, with nutrition data reported for women but not men. The Micronutrient Initiative details the number of women reached through its work. None of the partnerships which focus on the health of infants and children (e.g., through nutritional support or vaccines) provide sex-disaggregated data.

The 18 GPPPH ranged in focus from single issue (e.g., development of a vaccine) to population-wide (e.g., focus on health of mothers and children), with particular attention paid to infectious and communicable diseases in childhood. The majority of the initiatives (12/18) were concerned with the infectious and communicable diseases, three partnerships focused on nutrition (particularly malnutrition and deficiencies), and one was cross-cutting (Partnership for Maternal, Newborn and Child Health). None of the partnerships addressed the non-communicable diseases (NCDs) directly, but two of these addressed their determinants: the Global Alliance for Clean Cookstoves (focus on reducing indoor air pollution), and the Global Road Safety Partnership (working to reduce road-crash deaths and injuries).

## Discussion

Global public private partnerships in health have transformed the global health landscape over the past 20+ years, and the role of these partnerships has recently been strengthened with the adoption of the Agenda for Sustainable Development, which also commits the global community to achieving gender equality and empowerment of all women and girls (Goal 5). GPPPH are an important, although contested, component of the global response to contemporary global health challenges and several calls have been made to evaluate their effectiveness and the equity implications of the work they fund [38, 39] . Such evaluation, we argue, should include an analysis of how GPPPH perceive and address underlying social and structural determinants of health – including gender inequality and discrimination.

We found that gender is most often absent from the core strategies and policies of GPPPH, or, if present, suggest a wide variation in the understanding of gender - largely conceived as pertaining to women and girls. Such focus manifests in recognition of increased vulnerability of women and girls to ill-health (e.g., GAIN, Global Alliance for Clean Cookstoves, Micronutrient Initiative), women/girls’ lack of agency to redress a health problem (GAIN), or the higher care burden that often falls upon girls and women (Roll Back Malaria). Stop TB is one of the very few partnerships promoting the use of a gender-assessment tool to guide its work, yet its country level data is not disaggregated for gender specific differences in risks, health seeking and programme outreach. The Global Fund’s gender strategy is strong in its commitment to addressing gender inequalities that fuel the HIV epidemic (with a focus on women and girls), yet evaluation of its implementation and monitoring indicators suggests a major gap between policy intent and practice with too few grant agreements found to specify, fund or monitor gender-sensitive or transformative activities [40, 41].

The majority of GPPPH, however, are gender blind in their approach to health and lack simple mechanisms for enhancing gender accountability. Three notable omissions and gaps to gender transformative global health policies and programmes emerge from our analysis.

First, the vast majority of partnerships were governed by boards with unequal gender ratios (Table 1). The skewed composition of governing bodies of GPPPH, reflected in the conspicuous invisibility of women, reinforces concerns around tokenistic pursuit of goals of representation and rights by global health actors [42] and raises concerns for accountable governance in global health.

Second, the majority of GPPPH fail to report or publish sex-disaggregated data on coverage, outcomes or impact of the programmes they fund (Table 2). Where gender specific outcomes are reported, these are largely restricted to presenting what percentage of beneficiaries are women and girls. Such a view is not only limiting but may be counterproductive to tackling the underlying determinants of the global burden of disease. Sex and age data disaggregation on risk exposure, prevention and treatment coverage and outcomes are essential for understanding ill-health, ensuring investments are reaching those with highest need, and monitoring impact – including impact on reducing gender-based gaps in coverage and outcomes. Such information is vital to the work of ensuring that no-one is left behind in global health. For example, a systematic analysis of global incidence and mortality associated with HIV, TB and malaria over more than two decades found that mortality rates were higher in males than females for all three infections, while incidence rates were higher in females for malaria, higher in men for TB, and approximately equal for HIV [43] . A gendered interpretation of this picture may conclude that programmes concerned with gender norms around treatment seeking and health care coverage will need to include a focus on higher mortality rates in men (as an indicator of lower access to care) – but among our sample of partnerships, only Stop TB seems to be concerned with this dimension of gender. Holding GPPPH to account for gender and health outcomes means, at a minimum, having up-to-date sex-disaggregated data on coverage and outcomes.

Third, as shown in the two Figures, the partnerships have clustered the focus of their work on maternal health, child health and communicable/infectious diseases. The absence of any substantial body of GPPPH activity in addressing the highest burdens of disease (i.e., the non-communicable diseases and violence and injuries) represents a gross failure not only of evidence-informed resource allocation, but also a failure to recognize the gendered nature of health risks and suffering. Large proportions of the NCDs and their underlying risk behaviours, are currently more common in men and boys (e.g., diseases associated with tobacco, alcohol, occupational health exposures) [44] but rates of exposure are rising in women [45] – and will soon be followed by increased incidence of NCD-related ill-health. It has been argued (including by us [22]) that much of this risk behavior is influenced by profit-driven industries (e.g., consumption of tobacco [42], alcohol [43], highly processed foods [46]). Failure to address these areas signals a major abrogation of responsibility from the global health community. This lack of attention echoes the wider criticism of GHPPPs that the business-orientation endorsed by them has a bias for ‘safe issues’ [47] and narrow technical or ‘magic bullet’ approaches over tackling structural and more complex upstream determinants, including gender power relations [4, 15].

## Conclusions

The GPPPH are important players in global health. They constitute a major source of funding for health programmes in low- and middle-income countries, and exert influence over health decision-making at national and global levels. Therefore, understanding how the GPPPH address gender - a key determinant of health outcomes - raises legitimate issues of priority-setting, resource allocation and accountability.

Our finding of a widespread lack of a gender-aware approach within these partnerships is not unexpected, as similar results have been found in reviews of other major global health institutions [21, 22]. Nonetheless, gender equity should be central to the work that they do. Gender drives not only the risk of exposure to an illness-determinant, but also has a major influence over the likelihood that appropriate prevention, care and treatment services will be either sought, offered or received. Of equal and potentially greater concern, is the finding of an almost total absence of GPPPH activity to address the gendered nature of major health risks being faced by both women and men globally – in light of the growing burden of NCDs.

We believe that two lines of action are now needed. First, the existing GPPPH need to become more serious about how they “do gender”. It is not sufficient to mention girls and women in advocacy documents. Instead, a relational perspective on gender needs to be mainstreamed through the regular activities, deliverables and systems of accountability of all GPPPH – from boardroom to delivery/access to health services, gender needs to be fully taken into account.

Second the global health community needs to place much greater emphasis on tackling the major burdens of NCDs, including the gendered nature of risk for many of the NCDs. Whether GPPPH are the most appropriate model for tackling NCDs remains open to question. Given the key role that the private sector plays in determining the nature of risk (including manufacturing gendered risks) of exposure to health-reducing products, there is a strong argument for the need for interaction between global health communities and private companies. However, the broad yet specific challenge is how to manage the risks inherent in such interactions [48], mitigate conflicts of interest, and ensure that population health is protected [49] while also addressing the gendered nature of health determinants and health system responses.

## Footnotes

1 Data not included in the Table, but available
